# Effects of ambient climate and three warming treatments on fruit production in an alpine, subarctic meadow community

**DOI:** 10.1002/ajb2.1631

**Published:** 2021-03-31

**Authors:** Juha M. Alatalo, Annika K. Jägerbrand, Junhu Dai, Mohammad D. Mollazehi, Abdel‐Salam G. Abdel‐Salam, Rajiv Pandey, Ulf Molau

**Affiliations:** ^1^ Department of Biological and Environmental Sciences College of Arts and Sciences Qatar University P.O. Box 2713 Doha Qatar; ^2^ Environmental Science Center Qatar University P.O. Box 2713 Doha Qatar; ^3^ Calluna AB Hästholmsvägen 28 131 30 Nacka Sweden; ^4^ Department of Environmental and Biosciences Rydberg Laboratory of Applied Science (RLAS) School of Business, Engineering and Science Halmstad University P.O. Box 823 SE‐301 18 Halmstad Sweden; ^5^ Key Laboratory of Land Surface Pattern and Simulation Institute of Geographic Sciences and Natural Resources Research Chinese Academy of Sciences Beijing China; ^6^ University of Chinese Academy of Sciences Beijing China; ^7^ China‐Pakistan Joint Research Center on Earth Sciences CAS‐HEC Islamabad 45320 Pakistan; ^8^ Department of Mathematics, Statistics, and Physics College of Arts and Sciences Qatar University P.O. Box 2713 Doha Qatar; ^9^ Division of Forestry Statistics Indian Council of Forestry Research and Education Dehradun India; ^10^ Department of Plant and Environmental Sciences University of Gothenburg P.O. Box 461 SE‐405 30 Gothenburg Sweden

**Keywords:** climatic events, experimental warming, global warming, plant reproduction, polar region, rain fall, plant reproductive success, tundra

## Abstract

**Premise:**

Climate change is having major impacts on alpine and arctic regions, and inter‐annual variations in temperature are likely to increase. How increased climate variability will impact plant reproduction is unclear.

**Methods:**

In a 4‐year study on fruit production by an alpine plant community in northern Sweden, we applied three warming regimes: (1) a static level of warming with open‐top chambers (OTC), (2) press warming, a yearly stepwise increase in warming, and (3) pulse warming, a single‐year pulse event of higher warming. We analyzed the relationship between fruit production and monthly temperatures during the budding period, fruiting period, and whole fruit production period and the effect of winter and summer precipitation on fruit production.

**Results:**

Year and treatment had a significant effect on total fruit production by evergreen shrubs, *Cassiope tetragona*, and *Dryas octopetala*, with large variations between treatments and years. Year, but not treatment, had a significant effect on deciduous shrubs and graminoids, both of which increased fruit production over the 4 years, while forbs were negatively affected by the press warming, but not by year. Fruit production was influenced by ambient temperature during the previous‐year budding period, current‐year fruiting period, and whole fruit production period. Minimum and average temperatures were more important than maximum temperature. In general, fruit production was negatively correlated with increased precipitation.

**Conclusions:**

These results indicate that predicted increased climate variability and increased precipitation due to climate change may affect plant reproductive output and long‐term community dynamics in alpine meadow communities.

Climate change is already affecting plants by causing changes in phenology such as earlier flowering (Totland and Alatalo, 2002; Aerts et al., 2004; Høye et al., 2007; Beaubien and Hamann, 2011; Wang et al., 2014; Legault and Cusa, 2015), leafing out (Wipf, 2010; Zohner and Renner, 2014; Dai et al., 2019), delayed leaf senescence (Estiarte and Peñuelas, 2015; Gallinat et al., 2015; Yue et al., 2015; Liu et al., 2016) and delayed plant growth (Kudo et al., 1999; Campioli et al., 2013; De Long et al., 2015; Løkken et al., 2019; Villellas et al., 2019). In addition, climate change can affect flower, fruit, and seed production (Alatalo and Totland, 1997; Kudo and Suzuki, 2002; Kudo et al., 2004; Abeli et al., 2012; Panchen and Gorelick, 2015). It has also been shown to alter sex ratios between female and male plants, which in turn can affect reproductive success (Petry et al., 2016). Plant phenology and reproduction are important, as they affect tropic interactions (Aldridge et al., 2011; Liu et al., 2011; Høye et al., 2013; Kudo and Ida, 2013; Forrest, 2015; Gillespie et al., 2016). Previous climate change studies focusing on plant reproduction have used natural climate sequence data and analyzed the effect on reproduction (Molau, 1996; Inouye, 2008; Miller‐Rushing and Inouye, 2009; Abeli et al., 2012; Panchen and Gorelick, 2015) or have used experimental data (Aerts et al., 2004; Mallik et al., 2011; Liancourt et al., 2012; Liu et al., 2012; Semenchuk et al., 2013; Alatalo and Little, 2014). In most cases, these studies have focused on flower production (reproductive effort) (Inouye et al., 2002; Hollister et al., 2005; Semenchuk et al., 2013; Bienau et al., 2015), while fewer studies have examined fruit/seed production (reproductive success) (Totland and Alatalo, 2002; Mallik et al., 2011; Liu et al., 2012; Alatalo and Little, 2014; Panchen and Gorelick, 2015). The timing of flowering (phenology) can affect fruit production (reproductive success) (Hall et al., 2018). Seeds can also be sensitive to temperature, which can impact longevity, germination, and seedling survival (Bernareggi et al., 2015; Briceño et al., 2015). In addition, there may be complex interactions between the density of plant populations and their responses in terms of flowering phenology and fruit/seed production (Cao et al., 2016). Warming can also decrease nectar yield, thus negatively influencing pollinator interactions (Mu et al., 2015).

Alpine areas are predicted to be among the most vulnerable to future climate change. Plants in these harsh environments typically experience short summers, with weather conditions that are highly variable both within and between years. Flowering and fruit production are also dependent on weather conditions in the latter part of the previous season because many alpine and Arctic plant species initiate their flower buds in the year before actual flowering (Sørensen, 1941; Molau et al., 2005). Growing degree days have been shown to be important for phenology (White, 1979) and mean and minimum temperature for flowering abundance and seed set (Inouye et al., 2003; Kudo and Hirao, 2006). In addition, winter precipitation in cold alpine and Arctic areas frequently falls as snow. Increased winter precipitation could affect plant reproduction by affecting the timing of snowmelt, which affects flowering phenology (Bjorkman et al., 2015). Similarly, increased summer precipitation could negatively affect flowering and fruit production (Phoenix et al., 2001), which then affects plant reproductive strategy, as flowering plants have to cope with limited numbers of pollinators under unpredictable weather conditions (Totland, 1994; Lundemo and Totland, 2007). Thus, pollen limitation is common for alpine plants (Alatalo and Molau, 2001; Lundemo and Totland, 2007; Peng et al., 2014; Straka and Starzomski, 2015). One way for a species to cope with this limitation is to be self‐compatible. For example, a study in the subnival belt of the Hengduan Mountains, China, found that 97.1% of hermaphroditic species present were self‐compatible and that 88.2% showed autonomous or facilitated selfing (Peng et al., 2014). In addition, flower longevity often increases with elevation, extending the possibility of pollination (Trunschke and Stöcklin, 2017). Plants can also show high plasticity in their responses to environmental conditions and are thus able to respond in terms of increased growth or earlier flowering when favorable conditions occur (Dunne et al., 2003; Kudo and Hirao, 2006; Alatalo and Little, 2014).

Passive open‐top chambers (OTC) are commonly used to apply climate‐change treatments in plant ecological studies (Marion et al., 1997). The OTC simulates a static level of warming, which is not realistic for future climate change, which is expected to be increasingly variable between years. To date, there have been few multi‐approach climate‐change studies (Yang et al., 2018). It is currently unknown whether the impact of a single climate event differs from that of static warming (used in most temperature‐enhancement experiments) or from that of progressively increasing warming (Bjerke et al., 2011; Alatalo et al., 2014, 2016; Jägerbrand et al., 2014). Bender et al. (1994) originally used two types of experimental perturbations of temperature (press and pulse) to analyze population responses (Bender et al., 1994). Press warming disturbances are a more gradual or cumulative pressure, similar to a gradual or successive heating effect. Pulse warming may be explained as a temporary or relatively discrete disturbance. Pulse responses are expected to reflect adaptation to and recovery from extreme climate events. We therefore considered them to be suitable for the present study to analyze whether responses differed between the different temperature perturbations aimed at simulating constant increase in temperature, gradual warming, and single summer “heat wave”. Temperature treatments in the present analysis were control (static temperature during the experiment), press (a sequential increase in temperature), and pulse (a period of higher temperatures followed by ambient temperatures).

This experimental study is one of a series comparing the impact of singular warming events with those of static and progressive temperature enhancement. We previously reported on the impact of different temperature warming perturbations on growth and abundance of cryptogams and vascular plants (Alatalo et al., 2014, 2016). In the present study, we examined the impact of three kinds of temperature warming and ambient climate variables (maximum, minimum, and average temperature; winter and summer precipitation) on plant fruit production (reproductive success) in the plant community. The reason for including maximum temperature was that summer heatwaves are predicted to become more common in the future (Dosio et al., 2018), and it thus added a “natural” complement to the experimental pulse treatment. In addition, because the timing of flower bud initiation differs between species (previous year or present year), the impact of ambient climate parameters in the previous or present year can be expected to differ between broad functional plant groups. For example, evergreen and deciduous shrubs tend to initiate flower buds in the previous season, while graminoids and forbs tend to develop buds during the current year (Molau et al., 2005). Thus, evergreen and deciduous shrubs are more likely to be affected by the climate in the previous autumn than graminoids and forbs.

The following questions were addressed in terms of plant fruit production: (1) Are the responses to standard static OTC perturbations similar to those to press and pulse perturbations? (2) Are the responses to press and pulse perturbations significantly different from each other? (3) How do climate variables during different phases of fruit production (the year of flowering [“current”] and the year before flowering [“previous”]) affect fruit production of different functional plant groups? Experimental treatments consisted of (1) a static level of warming with open‐top chambers (increase ~1.9°C above ambient), (2) press warming, yearly stepwise increases in warming (by ~1.0, 1.9, and 3.5°C) and (3) pulse warming, a single first‐year pulse event of warming (increase ~3.5°C). Based on the reasoning above, we hypothesized that: (1) Warming has a positive effect on total fruit production, but the nature of the warming regime affects the response. (2) Stepwise warming (press warming) has greater effects on total fruit production in later study years than constant warming (OTC) or a single summer high level of warming (pulse warming). (3) Stepwise warming has the largest positive effect on fruit production in deciduous and evergreen shrubs, while pulse‐warming treatments have the largest positive effect on graminoids and forbs. (4) Total fruit production is positively correlated with temperature during the fruiting period (“current” year summer temperature), budding period (“previous” year autumn temperature), and the fruit production period (budding and fruit production combined). (5) Total fruit production is negatively correlated with winter precipitation during the winter before flowering and with summer precipitation during the fruiting period. (6) Fruit production by evergreen and deciduous shrubs is positively correlated with temperature during autumn of the previous year and summer of the current year, while fruit production of graminoids and forbs is affected more by temperature during summer of the current year.

## MATERIALS AND METHODS

The fieldwork was conducted in northernmost Sweden, at the Latnjajaure Field Station (LFS) in the Latnjavagge Valley (68°21′N, 18°29′E, 1000 m a.s.l.). Since early spring 1992, a year‐round automatic climate station has provided a continuous data set for the site.

The alpine valley is situated above the treeline and covered with snow for most of the year, and the climate is classified as subarctic, with cool summers, relatively mild, snow‐rich winters (annual minimum temperature ranging from −27.3 to −21.7°C) and mean annual temperature of −2.0 to −2.7°C (1993–1999). Annual precipitation ranges from 605 mm (1996) to 990 mm (1993), with a mean for 1990–1999 of 808 mm. July is the warmest month, with mean monthly temperature from +5.4°C (1992) to +9.9°C (1997).

The vegetation in the valley comprises a wide range of communities, varying from dry to wet and poor and acidic to base‐rich. Although the geographical situation is subarctic‐alpine, the vegetation of the area is representative of the Low Arctic, with *Cassiope tetragona* (Ericaceae), *Dryas octopetala* (Rosaceae), and *Carex bigelowii* (Cyperaceae) among the dominant species (Alatalo et al., 2016).

## Experimental design

The present experiment was set up in a rich meadow community around 300 m southeast of LFS, on a gentle, northwest‐facing slope with good groundwater supply (Molau and Alatalo, 1998). In July 1995, four blocks, each with four 1 × 1 m plots and as similar as possible with regard to floristic composition and edaphic conditions, were marked out and numbered. For main criteria, each plot had to have a medium‐sized tuft of the dwarf shrub *Cassiope tetragona* in its center and mesic, but not moist, soil conditions. Treatments were then allocated to plots within blocks by simple lottery by numbers.

**FIGURE 1:**
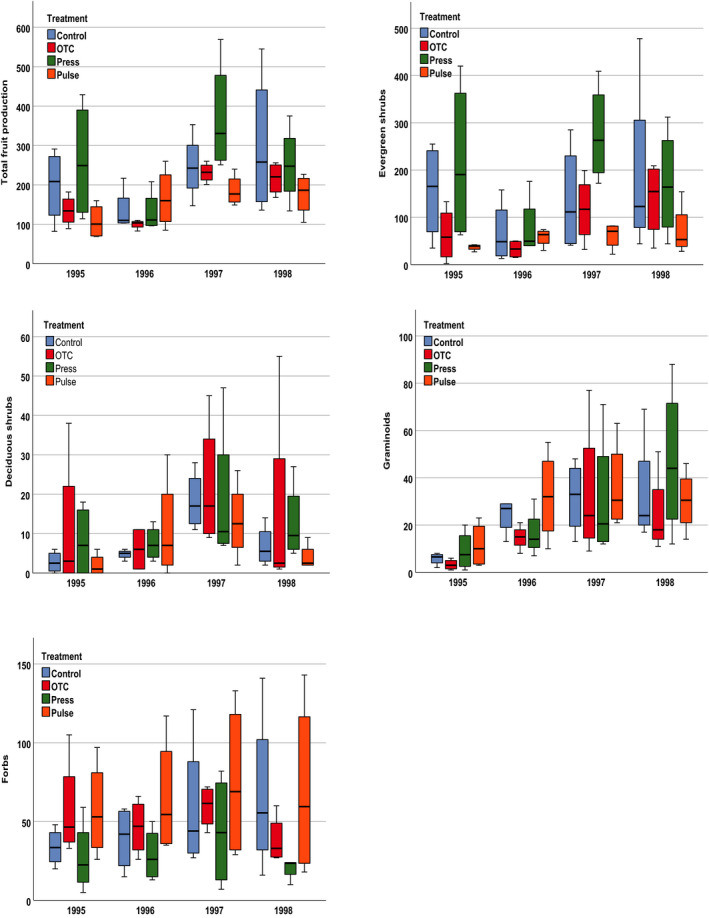
Response in terms of (top left) total fruit production (fruit production by all species) and (top right to bottom) fruit production by evergreen shrubs, deciduous shrubs, graminoids, and forbs across treatments in 1995, 1996, 1997, and 1998 in an alpine meadow community at Latnjajaure, northern Sweden. Treatments: control (Control), static warming enhancement with open‐top chambers (OTC), stepwise increasing magnitude of warming (Press) and a single‐summer high‐impact warming event (Pulse). Boxplots show the 10th to 90th percentile of the data; *N* = 4 plots per treatment.

At the end of the 1995 season, planned warming treatments were allocated within the blocks by simple lottery. Within each of the four blocks, four treatments were applied, starting in June 1996 (Fig. 1). These treatments were (1) control (with no temperature manipulation), (2) standard OTC, (3) press, and (4) pulse. In the standard OTC plots (treatment 2), hexagonal polycarbonate chambers (ITEX OTCs; International Tundra Experiment, https://www.gvsu.edu/itex/) with base diameter 1 m (Molau and Alatalo, 1998) were fixed to the ground from early June 1996 to late August 1998. In the press temperature manipulation plots (treatment 3), an OTC was installed in each plot on 10 cm high pegs throughout the 1996 season, affixed to the ground throughout the 1997 season, and fitted with a polyethylene lid throughout the 1998 season, thus increasing the experimental warming year‐on‐year (Alatalo et al., 2014). In the pulse plots (treatment 4), a closed‐top chamber (CTC; a standard OTC provided with a polyethylene lid as in treatment (3) was installed throughout the 1996 season only and removed in late August of the same year.

## Measurements

At the end of each season (late August, 1995–1998), the reproductive success of all vascular plant species was inventoried in all plots. Because we could not count all seeds from all species in all plots, we used the number of fruits, or infructescences (as in graminoids), as a proxy for reproductive success. While this count is not as accurate as actually counting all seeds produced by a plant, seed and fruit production have been shown to be positively correlated (Alatalo and Molau, 2001). The species were grouped into functional groups (evergreen shrubs, graminoids, deciduous shrubs, forbs, total fruit production) (Chapin et al., 1996).

Surface temperature in some of the treatment plots (always in comparison with parallel control plots) was measured with Tinytag temperature loggers (Gemini Data Loggers Ltd, Chichester, UK) recording at 30‐min intervals. The series from which means were calculated comprised 1000–5600 timed readings each. Although the weather conditions differed between the study years, the temperature increase brought about by the standard OTC remained relatively steady, at an average of 1.87 ± 0.25°C (mean ± SE, *N* = 7 runs) above the ambient (i.e., surface temperature in adjacent control plots). In the first treatment year, the ventilated OTCs in the press treatment resulted in a temperature increase of 1.00 ± 0.42°C (*N* = 2), while the CTC treatment in year 3 of the press treatment and in the 1‐year pulse treatment gave an increase of 3.54 ± 0.24°C (*N* = 3) above the control plots (Alatalo et al., 2014). The reference control plot surface temperature was on average 9.25 ± 0.55°C over the study seasons. Thus, the experimental temperature enhancement was classifiable into three temperature equivalents (units) of ~1°C each, where the cumulative warming after the entire experiment was equal for the OTC and press treatments, with a total of six units, whereas the pulse treatment received only three units above the control, although in one season (Alatalo et al., 2014).

## Statistical analyses

Most species (except for *Cassiope tetragona* and *Dryas octopetala*) were unevenly distributed among plots, preventing statistical analyses for comparison on species level. To test the importance of ambient climate variables for plants initiating flower buds at different times (previous year or current year), we grouped species into functional groups that broadly represented different strategies; deciduous and evergreen shrubs tend to initiate flower buds in the previous year, while graminoids and forbs tend to initiate flower buds during the current year (Molau et al., 2005).

To check for significant differences between treatments and years in the mean values of different response variables for individual species (*C. tetragona*, *D. octopetala*) and for functional plant groups (evergreen shrubs, graminoids, deciduous shrubs, forbs, total fruit production), we used generalized linear mixed model (GLMM), since it can include both fixed‐effect factors and within‐subject dependencies as random effects. We assumed that the block design (four blocks) could result in causality in the analyses, and we were not interested in analyzing block effects per se. Block design was therefore included as a random effect in the GLMM model and thereby treated as random variation around a population mean (Pinheiro and Bates, 2000). All data were transformed before analyses by ln (*c* + *x*) (where *x* is the response variable and *c* is a constant, −0.05 for this case) until skewness below 0.0001 was reached to ensure there was no heterogeneity or overdispersion, since that could influence the link function and normal distribution conditions. The following models were used in the GLMM: treatment, year, and treatment and year interactions (treatment × year) for four response variables. Response variables were fruits of *Cassiope tetragona*, evergreen shrubs, graminoids, deciduous shrubs, forbs, and total fruit production. Akaike’s information criterion (AIC) was used for evaluating the quality of fit of the models. Model settings were normal distribution and identity link function, while the build options were at default. As the data for *D*. *octopetala* were highly skewed even after transformation, we used the non‐parametric Kruskal‐Wallis test. Only the model with the best quality of fit is presented. In addition, we performed multiple comparisons (Bonferroni test) of the differences between treatments for all groups except *D. octopetala*.

The relationship between fruit production and ambient climate variables was estimated with Pearson’s correlation coefficient to examine the links between fruit production and temperature/precipitation. Mean monthly average temperature was considered along with mean maximum and monthly temperature, and monthly precipitation. The monthly temperature was considered in relation to the fruiting process, i.e., temperature of the months with flowering initiation and temperature of the months during fruit production. The flower initiation months were August, September, and October before fruit production year, i.e., in late summer–autumn of the previous year, which is called the budding period (Sørensen, 1941; Molau et al., 2005). The fruiting period months were May, June, July, and August in the current year. The budding period and fruiting period made up the fruit production period, which thus comprised 7 months, i.e., 3 months of budding period and 4 months of fruiting period. We estimated the mean maximum, minimum, and average temperature for these three periods, i.e., budding period, fruiting period, and fruit production period. The correlation between fruit production and temperature for all three periods was estimated for maximum (max) temperature, minimum (min) temperature, and average temperature of each month in the respective periods. The max, min, and average temperatures were used to capture potential effects of warm and cold events. Similarly, we estimated the effect of winter (October previous year to April current fruiting period), and summer (May to August of the fruiting period) precipitation on fruit production. The significance of correlation coefficients was assessed by a *t*‐test at 5% level of significance. All analyses were performed in IBM SPSS version 25 (IBM, Armonk, NY, USA).

## RESULTS

### Impact of experimental treatments on total fruit production

In terms of total reproductive success (fruit production), the plots and species assessed showed great individual variation. There was a significant effect of year and treatment (but not interaction) on total fruit production (Table 1), with large variations between treatments and years (Fig. 1; Appendix S1). However, multiple comparison tests found no significant difference between individual treatments (Appendix S2). The overall pattern across treatments was higher production of fruits in the press treatment than in the OTC and pulse treatments (Appendix S1). Among the study years, fruit production was poor in 1996 (following the cool summer of 1995) and higher in 1997 and 1998 compared with 1996 (Fig. 1).

**TABLE 1:** Type III tests of fixed effects from linear mixed models analysis, based on REML testing on the effects of year (1995, 1996, 1997, 1998) and treatment on total fruit production and on fruit production by evergreen shrubs, deciduous shrubs, graminoids, forbs, and *Cassiope tetragona* in an alpine meadow community at Latnjajaure, northern Sweden. Warming treatments: static warming enhancement with open‐top chambers (OTC), stepwise increasing magnitude of warming (Press) and a single‐summer high‐impact warming event (Pulse). Bold font on values indicates significance at *P* ≤ 0.05.

	df	*F*	*P*		df	*F*	*P*
**Total fruit production**				**Graminoids**			
Year	3	11.295	**0.000**	Year	3	21.226	**0.000**
Treatment	3	3.544	**0.022**	Treatment	3	2.027	0.124
Treatment × Year	9	1.247	0.292	Treatment × Year	9	0.539	0.838
**Evergreen shrubs**				**Forbs**			
Year	3	6.136	**0.001**	Year	3	0.994	0.404
Treatment	3	8.240	**0.000**	Treatment	3	7.164	**0.000**
Treatment × Year	9	1.453	0.195	Treatment × Year	9	0.424	0.915
**Deciduous shrubs**				***Cassiope tetragona***			
Year	3	6.834	**0.001**	Year	3	4.155	**0.011**
Treatment	3	0.792	0.505	Treatment	3	15.674	**0.000**
Treatment × Year	9	0.275	0.978	Treatment × Year	9	1.710	0.115

### Impact of experimental treatments on fruit production by plant functional groups

For the evergreen species as a group, there was a significant effect of both year and treatment (but not their interaction), with 1996 having the lowest numbers of fruits in all treatments except the pulse treatment, for which high‐level warming was applied in 1996 (Fig. 1, Table 1). Fruit production tended to be highest in the press treatment and lowest in the pulse and OTC treatments. Multiple comparison tests revealed a significant difference between the OTC and press, and the press and pulse treatments (Appendices S3 and S4).

There was a significant effect of year, but not treatment or interaction, on fruit production by deciduous shrubs (Fig. 1). There was no differential response to treatment, as fruit production by all species peaked in 1997 and then declined again in 1998 (Fig. 1, Table 1; Appendices S5 and S6).

There was a significant effect of year, but not treatment, on fruit production by the graminoid functional group (grasses and sedges) (Table 1). Fruit production increased across all treatments during the study period (Fig. 1), most likely as a result of the warm summers of 1996 and 1997. The control and press plots showed a steady increase in 1995–1998, whereas the OTC and pulse plots peaked in 1997 (Fig. 1). Fruit production was very similar across treatments (Appendix S7), and multiple comparison tests revealed no significant difference between individual treatments (Appendix S8).

In contrast, there was a significant treatment effect, but no effect of year or treatment × year interaction, on fruit production by forbs (Fig. 1, Table 1). Fruit production tended to be highest in the pulse treatment and lowest in the press treatment (Appendix S9). There was significantly lower fruit production in the press treatment compared with the control, OTC, and pulse treatments (Appendix S10). The responses varied widely between treatments and years (Fig. 1). Fruit production increased steadily in the control plots from 1995 to 1998 (Fig. 1). In the standard OTCs, there were no detectable trends in fruit production. The pattern that differed most markedly from the control plots was seen in the press treatment; fruit production increased in 1997 and then dropped in 1998 to a level below the initial (“before”) flowering of 1995.

**FIGURE 2:**
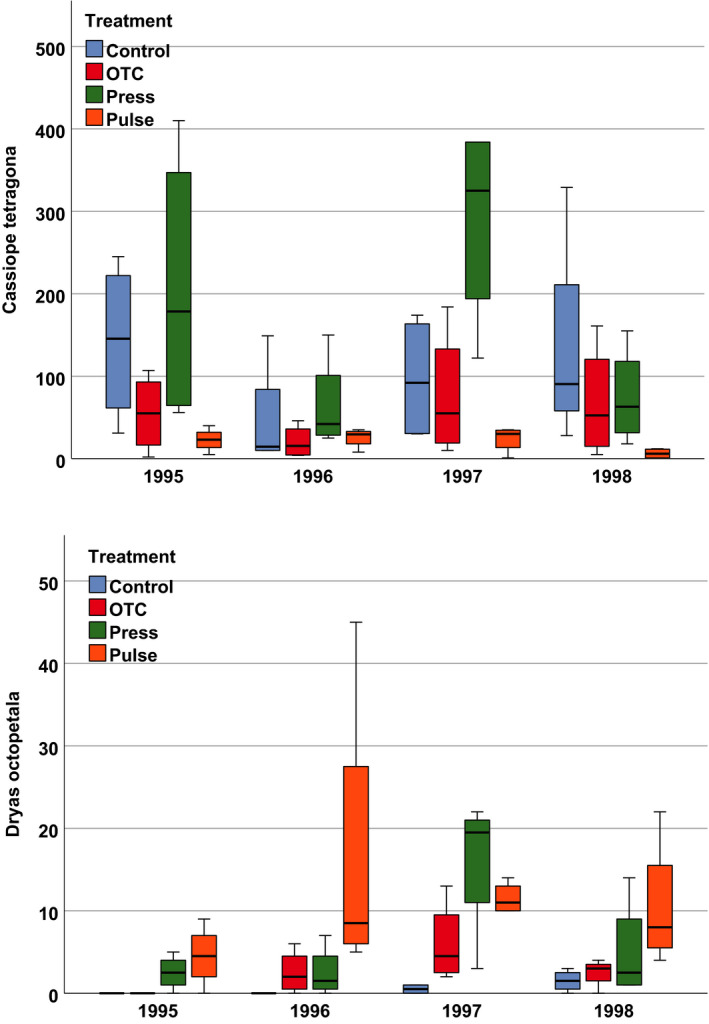
Responses in fruit production by (upper diagram) *Cassiope tetragona* and (lower diagram) *Dryas octopetala* across treatments in 1995, 1996, 1997 and 1998. Treatments: control (Control), static warming enhancement with open‐top chambers (OTC), stepwise increasing magnitude of warming (Press) and a single‐summer high‐impact warming event (Pulse). Boxplots show the 10th to 90th percentile of the data; *N* = 4 plots per treatment.

There was a significant effect of both year and treatment and a significant interaction between year and treatment on fruit production by *Cassiope tetragona* (Fig. 2, Table 1). Fruit production by this species tended to be highest in the press treatment and lowest in the pulse and OTC treatments (Appendix S11). There were significant differences between control and pulse, OTC and press, and press and pulse (Appendix S12). Total flowering in *C. tetragona* followed a similar pattern, with fruit production lowest in 1996 and higher in 1997 and 1998 (Fig. 2).

Similarly, there was a significant effect of both year and treatment (*p* = 0.013 and *p* = 0.000, respectively) on fruit production by *Dryas octopetala* (with pulse treatment having the highest and control the lowest fruit production). In this species, the pulse treatment induced a fruit production burst in 1996, which then slowly declined, while fruit production in the press treatment peaked in the second treatment year (1997) (Fig. 2; Appendix S13).

### Impact of ambient climate on fruit production

The correlation analysis showed that fruit production by *Cassiope tetragona* was positively correlated with mean maximum temperature for fruiting period and fruit production period, while it was negatively correlated with budding period (Table 2). Fruit production of *Dryas octopetala* was positively correlated for all three periods of fruit production with the minimum, maximum, and average temperature, except for maximum temperature in the budding period, which was negatively and not significantly correlated with maximum temperature in the full fruit production period (Table 2). Graminoid fruit production was positively correlated with minimum and average temperature for budding, fruit production, and the whole fruiting period (Table 2). Fruit production of deciduous shrubs was positively correlated with all three fruiting periods for minimum, maximum, and average temperature of the region, except for maximum temperature in the budding period (for which there was a negative correlation) (Table 2). Summer precipitation of the current fruit period had a negative effect on the fruit production of *D. octopetala*, graminoids, and deciduous shrubs (Table 3). Winter precipitation had a negative effect on fruit production of *C. tetragona* and all evergreen shrubs and a positive effect on graminoids (Table 3). While the combined precipitation (winter and summer) had a negative effect on fruit production of *D. octopetala*, deciduous and evergreen shrubs, and graminoids (Table 3).

**TABLE 2:** Correlation coefficients between fruit production and temperature in an alpine meadow community at Latnjajaure, northern Sweden (1995–1998). Budding period = August, September, and October before the fruit production year (i.e., previous year). Fruiting period = May, June, July, and August in the fruit production year (i.e., current year). Fruit production period = budding period + fruiting period (i.e., 7 months in total). Bold font on values indicates significance at *P* ≤ 0.05.

Variable	Total number of fruits produced (*p*)					
	*Cassiope tetragona*	*Dryas octopetala*	Evergreen shrubs	Graminoids	Deciduous shrubs	Forbs
Maximum temperature in budding period	−0.253 (**0.04**)	−0.224 (0.08)	−0.337 (**0.01**)	−0.167 (0.19)	−0.296 (**0.02**)	−0.114 (0.37)
Maximum temperature in fruiting period	0.264 (**0.04**)	0.215 (0.09)	0.265 (**0.03**)	0.119 (0.35)	0.336 (**0.00**)	0.142 (0.26)
Maximum temperature in fruit production period	0.224 (0.08)	0.157 (0.22)	0.113 (0.38)	0.029 (0.82)	0.320 (**0.01**)	0.150 (0.24)
Minimum temperature in budding period	0.065 (0.60)	0.413 (**0.00**)	0.274 (**0.03**)	0.599 (**0.00**)	0.471 (**0.00**)	0.127 (0.32)
Minimum temperature in fruiting period	0.113 (0.38)	0.399 (**0.00**)	0.252 (**0.04**)	0.526 (**0.00**)	0.501 (**0.00**)	0.154 (0.23)
Minimum temperature in fruit production period	0.083 (0.52)	0.411 (**0.00**)	0.269 (**0.03**)	0.579 (**0.00**)	0.486 (**0.00**)	0.137 (0.28)
Mean temperature in budding period	0.146 (0.25)	0.369 (**0.00**)	0.330 (**0.01**)	0.475 (**0.00**)	0.431 (**0.00**)	0.128 (0.31)
Mean temperature in fruiting period	0.056 (0.66)	0.402 (**0.00**)	0.176 (0.17)	0.562 (**0.00**)	0.512 (**0.00**)	0.153 (0.23)
Mean temperature in fruit production period	0.096 (0.45)	0.407 (**0.00**)	0.247 (**0.05**)	0.552 (**0.00**)	0.503 (**0.00**)	0.145 (0.24)

**TABLE 3:** Correlation coefficients between fruit production and precipitation in an alpine meadow community at Latnjajaure, northern Sweden (1995–1998). Winter precipitation = October–April before the fruit production year (i.e., previous winter). Summer precipitation = May, June, July, and August in the fruit production year (i.e., current year). Bold font on values indicates significance at *P* ≤ 0.05.

Variable	*Cassiope tetragona*	*Dryas octopetala*	Evegreen shrubs	Deciduous shrubs	Graminoids	Forbs	Total fruit production
Winter (W) precipitation	**−0.29**	0.01	**−0.22**	−0.05	**0.24**	−0.07	**−0.27**
Summer (S) precipitation	0.15	**−0.34**	−0.04	**−0.36**	**−0.64**	−0.07	**−0.18**
W + S precipitation	−0.02	**−0.42**	**−0.21**	**−0.49**	**−0.63**	−0.13	**−0.41**

## DISCUSSION

Our hypothesis that warming would have a positive effect on total fruit production was partly supported. As predicted functional plant groups and individual species studied displayed large variations in their responses to the different warming perturbations. There was a significant effect of experimental warming on total fruit production by evergreen shrubs, forbs, *Cassiope tetragona*, and *Dryas octopetala*, but not for graminoids and deciduous shrubs. Regarding the question of whether responses differed to static warming (standard OTC) and the press and pulse treatments, we found that for total fruit production, there was no significant difference between OTC and pulse warming. However, the press treatment gave higher total fruit production and, as hypothesized, the highest fruit production in later years of the experiment. Similarly, as hypothesized, evergreen shrubs and *C. tetragona* (an evergreen shrub) produced significantly more fruits in the press treatment than in OTC, whereas forbs produced significantly fewer fruits in the press treatment than in OTC. For graminoids and deciduous shrubs, there were no significant differences between OTC and the other warming treatments. Regarding the question of whether responses to the press and pulse treatment differed, evergreens and *C. tetragona* produced more fruits in the press compared with the pulse treatment, as hypothesized, and forbs showed the opposite response pattern, with pulse warming producing significantly more fruits than the press treatment. However, we found no difference in total fruit production. The largest effect was typically seen in the third year of the press treatment. The relatively few studies on climate change impacts on fruit production in alpine areas reported contrasting results. For example, a 4‐year study on 10 species in a subalpine meadow found that, while fruit production tended to be greater in warmed plots for most species, there was no significant effect for any species (Price and Waser, 1998). Another study found that warming had a negative effect on fruit production by *Silene acaulis* (Alatalo and Little, 2014). A study examining 3 years of warming in an alpine meadow in Tibet found contrasting effects on fruit production among the species present, e.g., no effect on *Kobresia pygmaea* or *Potentialla fruticosa*, a negative effect on *Astragalus rigidulus*, and a tendency for decreased fruit production in *Potentilla saundersiana* (Dorji et al., 2013). These responses of forb species were similar to those in the present study, i.e., with a negative effect of press temperature treatment and no effect of the other warming treatments on forbs. These contrasting results in terms of fruit production between evergreen shrubs and forbs may be due to differences in reproductive strategies between sites and species (Arft et al., 1999). For example, flowering and seed set by High Arctic populations of *D. octopetala* have been shown to increase rapidly in response to experimental warming, while *Empetrum hermaphroditum* is reported to show no response to warming (Wookey et al., 1993). High Arctic *C. tetragona* has been shown to make a trade‐off between allocation to reproductive effort and vegetative growth among years (Johnstone and Henry, 1997). In addition, plant reproductive success and long‐term community dynamics depend not only on the response of plants, but also on the response of potential pollinators to climate/temperature (Kudo et al., 2004; Høye et al., 2013; Kudo and Ida, 2013; Kudo, 2014). The importance of pollinators for community dynamics has been clearly shown in a study where experimentally decreasing pollinators caused a decline in both seedling diversity and abundance (Lundgren et al., 2016). Thus, while experimental warming may potentially create more favorable conditions for flower and fruit development, fruit development ultimately depends on whether the flowers are pollinated (self‐ or cross‐pollinated). The OTCs used in many studies may thus have a negative effect on pollination by limiting incoming pollen dispersed by wind and access by pollinators to flowers. However, while access may be limited, pollinators that arrive inside OTCs may potentially stay for longer within the warmer and partially enclosed OTC space. The CTCs used for the pulse and third‐year press treatments in the present study could potentially have a larger negative effect on access by pollinators. We did not see any clear evidence of this for the pulse treatment, which did not have lower fruit set than the other treatments in 1996. However, fruit production in the third‐year press treatment declined in all cases except for the graminoids, which increased their fruit production in 1998. Graminoids are in general wind‐pollinated, and we therefore expected the CTCs to have the largest negative effect on this group. However, in a study in Tibet, 97.1% of the alpine hermaphroditic plants studied were self‐compatible and had autonomous or facilitated selfing to a very large extent (Peng et al., 2014). Selfing may therefore have counteracted the limited access to external pollen in the press treatment in the present study.

While there are few directly comparable studies on fruit production, other measures have been used to assess reproductive success. A global meta‐analysis on the impact of short‐term warming on tundra plants using various measures of reproductive success (seed yield, seed mass, number of fruits, number of seeds/head, bulbil yield, bulbil mass, number of heads in fruits) found that short‐term warming tended to increase reproductive success throughout, but with colder sites having a larger positive response (Arft et al., 1999). Specifically, evergreen shrubs had a positive significant response in the fourth year, while forbs had a positive significant response to warming in the first year (Arft et al., 1999). However, in the present study, we found more complex responses of evergreen shrubs, e.g., the pulse treatment resulted in the fewest fruits, the press treatment in the most fruits. Studies on reproductive success focusing on seed production/mass/germination have found more consistent results, with warming increasing seed numbers in *Koenigia islandica* in an alpine meadow in Tibet (Cui et al., 2017), in *Rhodolirium montanum* in the Andes (Dudley et al., 2018), in *Silene acaulis* in alpine Sweden (Alatalo and Totland, 1997) and in *Ranunculus glacialis* in alpine Norway (Totland, 1999). Seed mass has also been shown to be positively affected by warming (Wookey et al., 1995; Totland and Alatalo, 2002; Cui et al., 2017), as has seed germination (Wookey et al., 1995). In contrast, a 3‐year study in the Tibetan plateau on nine multi‐flowered and three single‐flowered species found that warming had a negative effect on seed production per plant for all multi‐flowered species, but not for the single‐flowered species (Liu et al., 2012).

We found a significant effect of year on fruit production by the total plant community, evergreen shrubs, deciduous shrubs, graminoids, *C. tetragona*, and *D. octopetala*. Similarly, many studies have reported inter‐annual variation in reproductive success in alpine plant communities (Wagner and Mitterhofer, 1998; Kudo and Suzuki, 2002; Totland and Alatalo, 2002; Kudo and Hirao, 2006; Mizunaga and Kudo, 2017). Many of the plant species found at Latnjajaure initiate their flower buds in the year before actual flowering (Sørensen, 1941; Molau et al., 2005). Flower bud initiation broadly follows plant functional groups, so flowering and fruit production are also dependent on the weather conditions in the latter part of the previous season. The correlation analyses indicated that ambient temperature during the budding period, fruit production, and whole fruiting period had a significant impact on fruit production. However, the relative importance varied between species and functional groups. As hypothesized, maximum temperatures during the budding period in the previous year had a significant effect on fruit production of deciduous and evergreen shrubs, as these functional groups tend to initiate their flower buds in the latter part of the previous summer (Molau et al., 2005) and are expected to be favored by warm temperatures during the flower bud initiation period. However, we found no positive effect of current year maximum temperature on fruit production in graminoids and forbs, contradicting our hypothesis. Minimum and average ambient temperatures of the different fruit development periods more frequently had a significant impact on fruit production than maximum temperatures did, suggesting that short heat spells may be of less importance than cold spells. It is noteworthy that the only functional plant group for which we found no significant effect of ambient temperature on fruit production was forbs, perhaps because forbs are largely dependent on pollinators, whereas graminoids and deciduous shrubs are largely wind‐pollinated. Thus, forbs may be impacted by pollen limitation for fruit production due to the generally low abundance of pollinators in the harsh environment. *Cassiope tetragona* and *D. octopetala*, on the other hand, are known to be partially insect‐pollinated, as well as having the potential for self‐pollination (Kevan, 1972). Interestingly, *C. tetragona* and *D. octopetala* showed the opposite response patterns, i.e., fruit production by *C. tetragona* was significantly influenced by maximum temperatures during the budding and fruiting period, while fruit production by *D. octopetala* was influenced by minimum temperatures in these two periods. Overall, the favorable summers of 1996 and 1997 may have caused the majority of plant species to increase the number of flower buds, which in turn may have affected fruit production in the following years (1997 and 1998). There is evidence from experimental studies (Alatalo and Totland, 1997) and from studies using natural climate data (Molau et al., 2005) that the onset of reproductive phenology is temperature‐dependent. As in other studies, we found a significant negative effect of summer precipitation on fruit production by *D. octopetala*, graminoids and deciduous shrubs. Previous studies have shown that flowers of *Gentiana algida* that were experimentally forced to remain open during a rainfall event, experienced a substantial loss of pollen and reduced female fitness, affecting seed size, mass, number of ovules and viable seeds, and seed germination (Bynum and Smith, 2001). Similarly, 7 years of experimentally increased summer precipitation had a negative effect on flower and berry production of *Vaccinium myrtillus* in sub‐Arctic Sweden (Phoenix et al., 2001).

Winter precipitation in cold alpine areas frequently occurs as snowfall; thus, an increase in winter precipitation could cause a later onset of bare ground and subsequent delay in start of flowering, which could potentially decrease reproductive success. In line with this, we found a negative effect of increased winter precipitation on fruit production of *C. tetragona* and evergreen shrubs and a positive effect on graminoids. A transplant experiment simulating both earlier and delayed snowmelt in Norway showed high plasticity in the reproductive phenology of *Ranunculus acris* to the onset of snowmelt (Delnevo et al., 2018). However, a warming experiment resulted in contrasting responses in terms of reproductive phenology among plants on the Qinghai‐Xizang Plateau (Zhu, 2016). Snowmelt can be highly variable between years (Totland and Alatalo, 2002), and decreasing snow depth and earlier snowmelt have been shown to affect fruit production and seed set in a positive way (Alatalo and Totland, 1997; Bienau et al., 2014). However, the responses to snowmelt can be species‐specific and complex; e.g., earlier onset of snowmelt is reported to have a positive effect on flower production, but a negative effect on fruit production by *Salix herbacea* (Wheeler et al., 2016). A potential explanation for the contrasting flower/fruiting responses may be that earlier snowmelt is associated with greater exposure of bare plants to frost events (Wheeler et al., 2016). Thus, while plants may induce more flowers under earlier snowmelt, early season freezing events may cause more damage to the reproductive structures (Ladinig et al., 2013; Wheeler et al., 2016). In addition, climate change may enhance the potential for alien species to become invasive, as they can have greater phenological plasticity and increase their reproductive investment in response to simulated warming compared with native species (Cao et al., 2018). Moreover, as shown in this study, plant reproductive responses to increased variability in climate vary between species and warming patterns.

Changes in soil moisture could also influence plant reproductive success (Inouye et al., 2003; Dorji et al., 2013). For example, models based on experimental warming have been shown to underpredict the effect on timing of flowering, due to factors such as drier soils (Wolkovich et al., 2012). A meta‐analysis on the effect of experimental warming on soil moisture showed that the impact differed across ecosystems, with forests, grasslands, and croplands having significant declines, but with no significant effect on soil moisture in shrubland and tundra (Xu et al., 2013). While we did not measure soil moisture during our experiment, two decades of warming in a different experiment at the same site did not affect soil moisture in the organic soil layer (Alatalo et al., 2017). Thus, it is more likely that the amount of winter precipitation in the form of snow and early season temperature affect the timing of snowmelt and are thus the main drivers of soil moisture at the study site during the short growing season.

## CONCLUSIONS

In this experimental warming study, the reproductive success of alpine plant communities varied widely with year, experimental warming perturbation, functional plant groups, and species. As hypothesized, evergreen shrubs and forbs showed opposing responses to stepwise and pulse warming. In addition, fruit production was influenced by ambient temperature during the previous‐year budding period, current‐year fruit production period, and whole fruiting period. Minimum and average temperatures were more important than maximum temperatures, so periodic cold spells are likely to be more important than periodic warm spells. In addition, fruit production by most plant groups was in general negatively correlated with both winter and summer precipitation. The findings indicate a need to move forward with more multi‐faceted climate change experiments, rather than static warming treatments, to better simulate future increased climate variability. Fruit production by different plant groups responded differently to different climate perturbation treatments, which was partly explained by the timing of initiation of flower buds. The changes observed in fruit production indicate that both the predicted increased variability in climate and increased precipitation due to climate change are likely to affect long‐term community dynamics, which are influenced by both species diversity and abundance of seedlings.

## Author Contributions

U.M. designed the experiment; A.K.J., J.M.A., and U.M. carried out the fieldwork. J.M.A., A.K.J., M.D.M., S.G.A., and R.P. carried out the data analyses. A.K.J., J.M.A., and R.P. prepared the figures and tables. J.M.A. drafted the manuscript. All authors read, commented on, and approved the final manuscript.

## Supporting information


**APPENDIX S1.** Mean values of total fruit production in an alpine meadow community at Latnjajaure, northern Sweden.

**APPENDIX S2.** Multiple comparisons test by Bonferroni test (function ADJ that allows multiple comparisons by analyzing estimated marginal means) of the effect of treatment on total fruit production in an alpine meadow community at Latnjajaure, northern Sweden.

**APPENDIX S3.** Mean values of fruit production by evergreen shrubs in an alpine meadow community at Latnjajaure, northern Sweden.

**APPENDIX S4.** Multiple comparisons test by Bonferroni test (function ADJ that allows multiple comparisons by analyzing estimated marginal means) of the effect of treatment on fruit production by evergreen shrubs in an alpine meadow community at Latnjajaure, northern Sweden.

**APPENDIX S5.** Mean values of fruit production by deciduous shrubs in an alpine meadow community at Latnjajaure, northern Sweden.

**APPENDIX S6.** Multiple comparisons test by Bonferroni test (function ADJ that allows multiple comparisons by analyzing estimated marginal means) of the effect of treatment on fruit production by deciduous shrubs in an alpine meadow community at Latnjajaure, northern Sweden.

**APPENDIX S7.** Mean values of fruit production by graminoids in an alpine meadow community at Latnjajaure, northern Sweden.

**APPENDIX S8.** Multiple comparisons test by Bonferroni test (function ADJ that allows multiple comparisons by analyzing estimated marginal means) of the effect of treatment on fruit production by graminoids in an alpine meadow community at Latnjajaure, northern Sweden.

**APPENDIX S9.** Mean values of fruit production by forbs in an alpine meadow community at Latnjajaure, northern Sweden.

**APPENDIX S10.** Multiple comparisons test by Bonferroni test (function ADJ that allows multiple comparisons by analyzing estimated marginal means) of the effect of treatment on fruit production by forbs in an alpine meadow community at Latnjajaure, northern Sweden.

**APPENDIX S11.** Mean values of fruit production by *Cassiope tetragona* in an alpine meadow community at Latnjajaure, northern Sweden.

**APPENDIX S12.** Multiple comparisons test by Bonferroni test (function ADJ that allows multiple comparisons by analyzing estimated marginal means) of the effect of treatment on fruit production by *Cassiope tetragona* in an alpine meadow community at Latnjajaure, northern Sweden.

**APPENDIX S13.** Mean values of fruit production by *Dryas octopetala* in an alpine meadow community at Latnjajaure, northern Sweden.

**APPENDIX S14.** Data used for analyses.

## Data Availability

Data used for analyses are included in the supplementary materials listed in the Supporting Information section.
