# Supplementary material for: Effects of ambient climate and three warming treatments on fruit production in an alpine, subarctic meadow community
Source: Am J Bot. 2021 Mar 31;108(3):411–22. doi: 10.1002/ajb2.1631 (PMC8251864; doi:10.1002/ajb2.1631)
Supplement: Supplementary file 1 — APPENDIX S1. Mean values of total fruit production in an alpine meadow community at Latnjajaure, northern Sweden. [file AJB2-108-411-s006.docx]

**Appendix S1.** Mean values of total fruit production in an alpine meadow community at Latnjajaure, northern Sweden. Treatments: static warming enhancement with open-top chambers (OTC), stepwise increasing magnitude of warming (Press) and a single-summer high-impact warming event (Pulse). *N* = number of plots, SD = standard deviation.

| Total fruit production | | | |
| --- | --- | --- | --- |
| Treatment | Mean | *N* | SD |
| Control | 219.50 | 16 | 120.670 |
| OTC | 170.56 | 16 | 63.336 |
| Press | 253.25 | 16 | 138.131 |
| Pulse | 158.94 | 16 | 58.669 |
| Total | 200.56 | 64 | 106.062 |
