# Supplementary material for: Effects of ambient climate and three warming treatments on fruit production in an alpine, subarctic meadow community
Source: Am J Bot. 2021 Mar 31;108(3):411–22. doi: 10.1002/ajb2.1631 (PMC8251864; doi:10.1002/ajb2.1631)
Supplement: Supplementary file 3 — APPENDIX S3. Mean values of fruit production by evergreen shrubs in an alpine meadow community at Latnjajaure, northern Sweden. [file AJB2-108-411-s009.docx]

**Appendix S3.** Mean values of fruit production by evergreen shrubs in an alpine meadow community at Latnjajaure, northern Sweden. Treatments: static warming enhancement with open-top chambers (OTC), stepwise increasing magnitude of warming (Press) and a single-summer high-impact warming event (Pulse). *N* = number of plots, SD = standard deviation.

| Evergreen shrubs | | | |
| --- | --- | --- | --- |
| Treatment | Mean | *N* | SD |
| Control | 137.88 | 16 | 124.417 |
| OTC | 87.50 | 16 | 70.062 |
| Press | 185.63 | 16 | 132.371 |
| Pulse | 56.94 | 16 | 32.302 |
| Total | 116.98 | 64 | 108.252 |
