# Supplementary material for: Effects of ambient climate and three warming treatments on fruit production in an alpine, subarctic meadow community
Source: Am J Bot. 2021 Mar 31;108(3):411–22. doi: 10.1002/ajb2.1631 (PMC8251864; doi:10.1002/ajb2.1631)
Supplement: Supplementary file 5 — APPENDIX S5. Mean values of fruit production by deciduous shrubs in an alpine meadow community at Latnjajaure, northern Sweden. [file AJB2-108-411-s013.docx]

**Appendix S5.** Mean values of fruit production by deciduous shrubs in an alpine meadow community at Latnjajaure, northern Sweden. Treatments: static warming enhancement with open-top chambers (OTC), stepwise increasing magnitude of warming (Press) and a single-summer high-impact warming event (Pulse). *N* = number of plots, SD = standard deviation.

| Deciduous shrubs | | | |
| --- | --- | --- | --- |
| Treatment | Mean | *N* | SD |
| Control | 8.13 | 16 | 7.562 |
| OTC | 13.56 | 16 | 17.466 |
| Press | 11.75 | 16 | 11.642 |
| Pulse | 7.56 | 16 | 9.077 |
| Total | 10.25 | 64 | 12.020 |
