# Supplementary material for: Effects of ambient climate and three warming treatments on fruit production in an alpine, subarctic meadow community
Source: Am J Bot. 2021 Mar 31;108(3):411–22. doi: 10.1002/ajb2.1631 (PMC8251864; doi:10.1002/ajb2.1631)
Supplement: Supplementary file 7 — APPENDIX S7. Mean values of fruit production by graminoids in an alpine meadow community at Latnjajaure, northern Sweden. [file AJB2-108-411-s008.docx]

**Appendix S7.** Mean values of fruit production by graminoids in an alpine meadow community at Latnjajaure, northern Sweden. Treatments: static warming enhancement with open-top chambers (OTC), stepwise increasing magnitude of warming (Press) and a single-summer high-impact warming event (Pulse). N = number of plots, SD = standard deviation.

| Graminoids | | | |
| --- | --- | --- | --- |
| Treatment | Mean | N | SD |
| Control | 23.75 | 16 | 17.430 |
| OTC | 19.00 | 16 | 19.667 |
| Press | 25.88 | 16 | 24.953 |
| Pulse | 27.56 | 16 | 17.255 |
| Total | 24.05 | 64 | 19.851 |
