# Supplementary material for: Effects of ambient climate and three warming treatments on fruit production in an alpine, subarctic meadow community
Source: Am J Bot. 2021 Mar 31;108(3):411–22. doi: 10.1002/ajb2.1631 (PMC8251864; doi:10.1002/ajb2.1631)
Supplement: Supplementary file 9 — APPENDIX S9. Mean values of fruit production by forbs in an alpine meadow community at Latnjajaure, northern Sweden. [file AJB2-108-411-s004.docx]

**Appendix S9.** Mean values of fruit production by forbs in an alpine meadow community at Latnjajaure, northern Sweden. Treatments: static warming enhancement with open-top chambers (OTC), stepwise increasing magnitude of warming (Press) and a single-summer high-impact warming event (Pulse). *N* = number of plots, SD = standard deviation.

| Forbs | | | |
| --- | --- | --- | --- |
| Treatment | Mean | *N* | SD |
| Control | 49.75 | 16 | 35.343 |
| OTC | 50.50 | 16 | 20.935 |
| Press | 30.00 | 16 | 22.724 |
| Pulse | 66.88 | 16 | 41.617 |
| Total | 49.28 | 64 | 33.325 |
