# Supplementary material for: Effects of ambient climate and three warming treatments on fruit production in an alpine, subarctic meadow community
Source: Am J Bot. 2021 Mar 31;108(3):411–22. doi: 10.1002/ajb2.1631 (PMC8251864; doi:10.1002/ajb2.1631)
Supplement: Supplementary file 10 — APPENDIX S10. Multiple comparisons test by Bonferroni test (function ADJ that allows multiple comparisons by analyzing estimated marginal means) of the effect of treatment on fruit production by forbs in an alpine meadow community at Latnjajaure, northern Sweden. [file AJB2-108-411-s007.docx]

**Appendix S10.** Multiple comparisons test by Bonferroni test (function ADJ that allows multiple comparisons by analyzing estimated marginal means) of the effect of treatment on fruit production by forbs in an alpine meadow community at Latnjajaure, northern Sweden. Treatments: static warming enhancement with open-top chambers (OTC), stepwise increasing magnitude of warming (Press) and a single-summer high-impact warming event (Pulse). SE = standard error.

| (I) Treatment | | Mean Difference (I-J) | SE | df | Sig. ^a^ | 95% Confidence Interval for Difference^b^ | |
| --- | --- | --- | --- | --- | --- | --- | --- |
|  |  |  |  |  |  | Lower Bound | Upper Bound |
| Control | OTC | -0.102 | 0.150 | 57.000 | 1.000 | -0.513 | 0.308 |
|  | Press | .439^*^ | 0.150 | 57.000 | **0.030** | 0.029 | 0.849 |
|  | Pulse | -0.255 | 0.150 | 57.000 | 0.570 | -0.665 | 0.155 |
| OTC | Control | 0.102 | 0.150 | 57.000 | 1.000 | -0.308 | 0.513 |
|  | Press | .541^*^ | 0.150 | 57.000 | **0.004** | 0.131 | 0.952 |
|  | Pulse | -0.152 | 0.150 | 57.000 | 1.000 | -0.563 | 0.258 |
| Press | Control | -.439^*^ | 0.150 | 57.000 | **0.030** | -0.849 | -0.029 |
|  | OTC | -.541^*^ | 0.150 | 57.000 | **0.004** | -0.952 | -0.131 |
|  | Pulse | -.694^*^ | 0.150 | 57.000 | **0.000** | -1.104 | -0.283 |
| Pulse | Control | 0.255 | 0.150 | 57.000 | 0.570 | -0.155 | 0.665 |
|  | OTC | 0.152 | 0.150 | 57.000 | 1.000 | -0.258 | 0.563 |
|  | Press | .694^*^ | 0.150 | 57.000 | **0.000** | 0.283 | 1.104 |
| Based on estimated marginal means. | | | | | | | |
| ^a^Adjustment for multiple comparisons: Bonferroni. | | | | | | | |
