# Supplementary material for: Effects of ambient climate and three warming treatments on fruit production in an alpine, subarctic meadow community
Source: Am J Bot. 2021 Mar 31;108(3):411–22. doi: 10.1002/ajb2.1631 (PMC8251864; doi:10.1002/ajb2.1631)
Supplement: Supplementary file 11 — APPENDIX S11. Mean values of fruit production by Cassiope tetragona in an alpine meadow community at Latnjajaure, northern Sweden. [file AJB2-108-411-s014.docx]

**Appendix S11.** Mean values of fruit production by *Cassiope tetragona* in an alpine meadow community at Latnjajaure, northern Sweden. Treatments: static warming enhancement with open-top chambers (OTC), stepwise increasing magnitude of warming (Press) and a single-summer high-impact warming event (Pulse). *N* = number of plots, SD = standard deviation.

| *Cassiope tetragona* | | | |
| --- | --- | --- | --- |
| Treatment | Mean | *N* | SD |
| Control | 105.06 | 16 | 95.277 |
| OTC | 54.69 | 16 | 56.611 |
| Press | 158.56 | 16 | 140.306 |
| Pulse | 19.63 | 16 | 13.861 |
| Total | 84.48 | 64 | 102.237 |
