# Supplementary material for: Effects of ambient climate and three warming treatments on fruit production in an alpine, subarctic meadow community
Source: Am J Bot. 2021 Mar 31;108(3):411–22. doi: 10.1002/ajb2.1631 (PMC8251864; doi:10.1002/ajb2.1631)
Supplement: Supplementary file 13 — APPENDIX S13. Mean values of fruit production by Dryas octopetala in an alpine meadow community at Latnjajaure, northern Sweden. [file AJB2-108-411-s003.docx]

**Appendix S13.** Mean values of fruit production by *Dryas octopetala* in an alpine meadow community at Latnjajaure, northern Sweden. Treatments: static warming enhancement with open-top chambers (OTC), stepwise increasing magnitude of warming (Press) and a single-summer high-impact warming event (Pulse). *N* = number of plots, SD = standard deviation.

| *Dryas octopetala* | | | |
| --- | --- | --- | --- |
| Treatment | Mean | *N* | SD |
| Control | 0.50 | 16 | 0.894 |
| OTC | 2.75 | 16 | 3.435 |
| Press | 6.50 | 16 | 7.677 |
| Pulse | 10.81 | 16 | 10.394 |
| Total | 5.14 | 64 | 7.636 |
